# China’s greenhouse gas emissions for cropping systems from 1978–2016

**DOI:** 10.1038/s41597-021-00960-5

**Published:** 2021-07-13

**Authors:** Dijuan Liang, Xi Lu, Minghao Zhuang, Guang Shi, Chengyu Hu, Shuxiao Wang, Jiming Hao

**Affiliations:** 1 Beijing Laboratory of Environmental Frontier Technologies, School of Environment, Tsinghua University, Beijing, 100084 China; 2 State Key Joint Laboratory of Environment Simulation and Pollution Control and State Environmental Protection Key Laboratory of Sources and Control of Air Pollution Complex, Tsinghua University, Beijing, 100084 P. R. China; 3 College of Resources and Environmental Sciences; National Academy of Agriculture Green Development, Key Laboratory of Plant-Soil Interactions of MOE, China Agricultural University, Beijing, P.R. China; 4 Department of Environmental Health, Harvard T.H. Chan School of Public Health, Boston, MA USA; 5 School of Computer Science, China University of Geosciences, Wuhan, 430074 China

**Keywords:** Agriculture, Climate change

## Abstract

China has committed to reaching carbon neutrality by 2060, which will require a drastic cut in greenhouse gas (GHG) emissions from all sectors, including those from agricultural activities. A comprehensive, long-term, and spatially-precise profile of agricultural GHG emissions can help to accurately understand drivers of historical emissions and their implications for future mitigation. This study constructs province-level agricultural GHG emissions in China from 1978 to 2016. It considers primary and secondary emissions from a full range of agricultural activities related to crop farming, including crop residue open burning, rice cultivation, cropland change, cropland emissions, machinery use, nitrogen fertilizer production, and pesticide production. Annual or interpolated activity data from official sources and the latest emission factors available for China were adopted in this study. The data can be used in spatial and temporal analysis of emissions from cropping systems as well as the design of mitigation strategy in China.

## Abstract (summary)

**Table:** 

Measurement(s)	greenhouse gas
Technology Type(s)	digital curation • computational modeling technique
Factor Type(s)	date • province
Sample Characteristic - Environment	cultivated environment • climate change
Sample Characteristic - Location	China

Machine-accessible metadata file describing the reported data: 10.6084/m9.figshare.14753940

## Background & Summary

According to the Synthesis Report (SYR) of the IPCC Fifth Assessment Report (AR5)^1^, agriculture, forestry, and other land use (AFOLU) contributed around 25% of total anthropogenic greenhouse gas (GHG) emissions globally in 2010. In particular, agricultural activities were the key sources for emissions of the non-CO_2_ GHG including CH_4_ and N_2_O, which have global warming potentials (GWP), respectively, of 28 and 265 times the value of CO_2_ on a 100 year time scale^2^.

With 18% of the global population^3^, China had more than 367 million people working in agriculture in 2019^4^. Changes in agricultural activities caused by urbanization in turn pose challenges for GHG emission mitigation in agriculture. Urbanization in China has developed rapidly with a continuous decrease in the rural population. The percentage of agricultural labor decreased from 83.5% in 1952 to 25.1% in 2019^5^. The Reform and Opening policy adopted in 1978 accelerated this process by encouraging agricultural labor to move to cities for jobs with higher salaries. To ensure crop production, agricultural activities have become more and more reliant on fertilizers and machinery inputs rather than labor and land. The use of fertilizers, pesticides, and machinery power has increased rapidly since 1978^5^. The total power of agricultural machinery reached 1027 GW in 2019^5^, over 8 times the value in 1978^5^. China peaked in fertilizer and pesticide use in 2017^6^. Associated with urbanization and agricultural modernization, the use of machinery is expected to continue increasing^4,7^. The GHG emissions associated with this increasing use of machinery raise challenges in agricultural mitigation in China. In 2012, although crop farming and animal husbandry only contributed 7.8% of total GHG emissions in China, they contributed over 50% of total non-CO_2_ emissions^8^. To realize carbon neutrality in China by 2060^9^, GHG emission mitigation in agriculture will be an important target^10^.

A number of studies have investigated long-term GHG emissions in the U.S. and globally^11–13^. Based on Tier 1 IPCC Guidelines^14^, FAOSTAT calculated 1961–2017 GHG emissions from agriculture, land use and forestry for 234 countries, which included emissions from enteric fermentation, rice cultivation, agricultural soil, crop residues and savanna burning, and energy use in agricultural activities^11^. Burney *et al*.^12^ calculated global agricultural GHG emissions from 1961 to 2005, which included emissions from agricultural soils, rice cultivation, cropland expansion, and production and use of fertilizers. Parton *et al*.^13^ used ecosystem models to estimate GHG emissions from agriculture from 1870 to 2000 in the U.S. Great Plains, including emissions from soil cultivation, N mineralization, N fertilizer application, livestock, fossil fuels used in fertilizer production, and burning of fossil fuels by farm equipment.

A holistic and integrated database for provincial agricultural GHG emissions is still lacking in China. Based on IPCC Guidelines^14^, China published official GHG inventories for 1994^15^, 2005^16^, and 2012^8^, in which enteric fermentation, manure management, rice cultivation, cropland soil, agricultural wastes burning, and land-use change were included for agricultural and land-use emissions^8,15,16^. Zuo *et al*.^17^ calculated GHG emissions from changes in crop mix, multiple cropping, fertilizer application, irrigation, and paddy rice water management in China, but only presented emissions for 1987, 2000, and 2010 rather than a continuous result. However, those studies selected different agricultural activities and parameters, which made it difficult for comparison. In addition, agricultural machinery uses and emissions from fertilizer and pesticide production were excluded from those studies. Most importantly, the existing datasets could not offer the GHG emissions profiles for agriculture in China either for the long-term perspective or on the provincial level and thus had limited application in designing mitigation strategies.

To fill those gaps, this study builds a database for provincial agricultural GHG emissions in China from 1978–2016. It covers crop residue open burning, rice cultivation, cropland change, cropland emissions, machinery use, nitrogen fertilizer production, and pesticide production. Up-to-date annual activity data was used for most agricultural activities and numerical interpolation was adopted for cropland change. Our analysis selected the latest emission factors available for China. Providing a complete and continuous profile of agricultural GHG emissions in China at the provincial level, this study can aid further analysis of contributions from different activities as well as spatial and temporal emission patterns, which are the foundation for designing mitigation strategies.

## Methods

Agricultural GHG emissions can be categorized into three types of sources: primary, secondary, and tertiary^18^. Primary emissions are defined as GHG emissions coming directly from tillage, seeding, harvesting and transportation^18^. Secondary emissions refer to GHG emissions from the production, packaging, and storage of fertilizers and pesticides^18^. Tertiary emissions denote GHG emissions from the acquisition of raw materials, manufacture of agriculture equipment like agriculture machinery, and construction of agricultural buildings^18^. According to the IPCC definition, primary emissions are direct emissions, and secondary and tertiary emissions are defined as embodied emissions and indirect emissions^19^. Primary emissions gain wide attention in GHG inventory compilation and carbon management. Agricultural activities mainly emit CO_2_, CH_4_, and N_2_O in primary emissions through livestock enteric fermentation, manure management, rice cultivation, cropland, crop residue burning, energy use, and land cultivation^8,14,20^. Secondary emissions are often accounted for in the life cycle analysis (LCA) of agricultural products. Since the boundary of tertiary emissions is broad, most studies concentrated on the primary and secondary emissions to guide the practice of agricultural GHG control^11,13,17^.

The study mainly focuses on crop farming and covers primary and secondary emissions. For primary emissions, the study included crop residue open burning, rice cultivation, cropland change, and cropland emissions based on China’s official inventory in 2012^8^. For secondary emissions, we considered machinery use, N fertilizer production, and pesticide production based on FAO2014^20^. Crop residue open burning can emit CO_2_, CH_4_, and N_2_O^14^, however only CH_4_ and N_2_O emissions are taken into account as CO_2_ released from burning is countered by carbon uptake from the air during the growth of crops. During rice cultivation, the anaerobic decomposition of organic materials in inundated rice fields emits CH_4_^14^. Emissions from cropland change can be positive or negative, accounting for CO_2_ emission or absorption due to the release or absorption of carbon by vegetation and soil during land-use changes between cropland and other land-use types like wood, grassland, and residential land^14^. Application of synthetic nitrogen fertilizers, manure, and crop residue on land directly emits N_2_O through nitrification and denitrification, and indirectly emits N_2_O through volatilization and leaching^20–22^. Agricultural machinery use consumes energy like diesel, gasoline, and electricity, which emits CO_2_, CH_4_, and N_2_O emissions^20^. Production of nitrogen fertilizers and pesticides produces CO_2_, CH_4,_ and N_2_O emissions during manufacture and transportation.

**Fig. 1:**
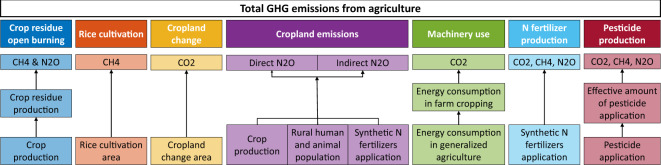
Diagram of the evaluation method framework.

The study included 31 provinces in mainland China (excluding Hong Kong Special Administrative Region, Macao Special Administrative Region, and Taiwan province). Since Hainan province was founded in 1987 and Chongqing city was founded in 1997, their emissions before establishment were calculated under Guangdong province and Sichuan province respectively. The following GHG emissions were counted as CO_2_ equivalent, and Global Warming Potential (GWP) values for the 100-year time horizon published by IPCC Fifth Assessment Report (AR5) in 2013 were adopted to transform CH_4_ (GWP_100_ = 28) and N_2_O (GWP_100_ = 265) emissions^2^. Total emissions calculated with the GWP values for the 20-year time horizon were included in the Supplementary Information (Figure S1 and Table S11). Missing activity values in certain years were linearly interpolated from the available values of the closest years. If there was only one close year with existing activity value, missing activity values in those years were taken as the available values in that closest year. The flowchart of the method of this study is illustrated in Fig. 1. The values and units of parameters used in the following calculation are included in Table 1.

**Table 1:** Value, coefficient of variation (CV) or range of distribution of parameters.

Agricultural activity	Parameter	Value	CV or (a, b)
Crop residue open burning	${f}_{s}^{str}$	Table S1	Table S1
	${f}_{i}^{open}$	Table S2	Table S2
	*f*^ *burn*^	0.9	0%
	*EF*^*CROP*^ for CH_4_	3.23 g CH_4_/kg	17%
	*EF*^*CROP*^ for N_2_O	0.008 g N_2_O/kg	23%
Rice cultivation	${f}_{i,r}^{length}$, $E{F}_{i,r}^{RICE}$	Table S3	Table S3
Cropland change	$E{F}_{j,l}^{LAND}$	Table S4	Table S4
Cropland emission	*f*^ *enn*^	0.2	0%
	${f}_{a}^{excr}$, ${f}_{a}^{mana}$, ${f}_{a}^{liq}$, ${f}_{a}^{sol}$	Table S5	Table S5
	${f}_{s}^{retu}$	0.14	11%
	${f}_{s}^{dry}$, ${f}_{s}^{rb}$, ${f}_{s}^{harv}$, ${f}_{s}^{rbrate}$	Table S6	Table S6
	$E{F}_{i}^{CRLA,d}$	Table S7	Table S7
	*f*^ *Fvolat*^	0.1 kg NH3-N and NOX-N/kg N applied	(0.03, 0.3)
	*f*^ *Ovolat*^	0.2 kg NH3-N and NOX-N/kg N applied	(0.05, 0.5)
	*EF*^*CRLA,atm*^	0.01 kg N_2_O-N/kg NH3-N and NOx-N volatilzed	(0.002, 0.05)
	*f*^ *leach*^	0.3 kg N/kg N additions or deposition by grazing animals	(0.1, 0.8)
	*EF*^*CRLA,leach*^	0.0075 kg N_2_O-N/kg N leaching or runoff	(0.0005, 0.025)
Machinery use	${f}_{e}^{LCV}$, ${f}_{e}^{carb}$, ${f}_{e}^{oxid}$	Table S8	Table S8
	$E{F}_{j,e}^{ENER}$	Table S9	Table S9
Nitrogen fertilizer production	*EF*^*FERT*^ for CO_2_	6.08 kg CO_2_/kg N	50%
	*EF*^*FERT*^ for CH_4_	0.02 kg CH_4_/kg N	50%
	*EF*^*FERT*^ for N_2_O	0.0009 kg N_2_O /kg N	50%
Pesticide production	${f}_{j}^{plPest}$	Table S10	50%
	*EF*^*PEST*^	16.35 kg CO_2_-equivalent/kg application	50%

### Crop residue open burning

According to Lu *et al*.^23^, CH_4_ and N_2_O emissions from crop residue open burning were calculated through Eq. (1):

1 $${E}_{i,j}^{CROP}=\left(\mathop{\sum }\limits_{s=1}^{13}Pro{d}_{i,j,s}\times {f}_{s}^{str}\right)\times {f}_{i}^{open}\times {f}^{burn}\times E{F}^{CROP}$$

where ${E}_{i,j}^{CROP}$ is crop residue open burning GHG emissions from province *i* in year *j*; *Prod*_*i,j,s*_ is the production for crop type *s* in the corresponding province and year; ${f}_{s}^{str}$ is the straw to grain ratio for the corresponding crop type; ${f}_{i}^{open}$ is the open burning ratio for the corresponding province; *f*^ *burn*^ is the burning efficiency; *EF*^*CROP*^ is the emission factor.

The study considered 13 types of crops, including rice, wheat, corn, millet, jowar, other cereal, beans, tubers, cotton, oil-bearing crops, fiber crops, sugar crops, and tobacco. Annual crop production data for each province was collected from the National Bureau of Statistics of China (NBSC)^5^. Straw to grain ratios were derived from the mean values of studies in China^23–27^. The open burning ratios for different provinces were calculated from the mean value of two studies in China^28,29^. The study adopted the burning efficiency recommended by Guidelines for Compiling Biomass Burning Air Pollutants Emission Inventories (Trial)^30^ published by the Ministry of Ecology and Environment of the People’s Republic of China in 2015. CH_4_ emission factor was developed from the mean value of EPA1992 Open Burning table^31^, Andreae *et al*.^32^, IPCC Guidelines^14^, and the three most recent studies in China^28,29,33^. N_2_O emission factor was collected from the mean value of Andreae *et al*.^32^, IPCC 2006 Guidelines^14^, and one study in China^33^.

### Rice cultivation

According to Yan *et al*.^34^, CH_4_ emissions from rice cultivation were calculated as in Eqs. (2) and (3):

2 $${E}_{i,j}^{RICE}=\mathop{\sum }\limits_{r=1}^{3}R{A}_{i,j,r}\times {f}_{i,r}^{length}\times E{F}_{i,r}^{RICE}$$

3 $$E{F}_{r}^{RICE}=\frac{1}{2}\times \left(\frac{1}{3}\times E{F}_{r}^{F\& CI}+\frac{2}{3}\times E{F}_{r}^{F\& SI}+\frac{1}{3}\times E{F}_{r}^{NF\& CI}+\frac{2}{3}\times E{F}_{r}^{NF\& SI}\right)$$

where ${E}_{i,j}^{RICE}$ is rice cultivation GHG emissions from province *i* in year *j*; *RA*_*i,j,r*_ is the cultivation area of rice type *r* in the corresponding province and year; ${f}_{i,r}^{length}$ is the length of the rice-growing period of rice type *r* in the corresponding province; $E{F}_{i,r}^{RICE}$ is the emission factor of the corresponding rice type and province, which is the weighted average of emission factor with organic output and continuous flooding ($E{F}_{r}^{F\& CI}$), emission factor with organic output and intermittent irrigation ($E{F}_{r}^{F\& SI}$), emission factor without organic output but with continuous flooding ($E{F}_{r}^{NF\& CI}$), and emission factor without organic output but with intermittent irrigation ($E{F}_{r}^{NF\& SI}$). Following the study by Yan *et al*.^34^, we assumed 1/2 of the rice cultivation area had organic input, 1/3 of the rice cultivation area had continuous flooding, and 2/3 rice cultivation area had intermittent irrigation.

The present study considered 3 rice types, including early rice, middle rice and single late rice, and double late rice. The annual rice cultivation area for different provinces was collected from annual data from the NBSC^5^. The length of the rice-growing period and emission factors of each rice type and province were obtained from Yan *et al*.^34^. For Shanghai, Jiangsu, Anhui, Henan, and Hubei (Agroecological zones AEZ 6 A regions), we adopted the mean values of emission factors of middle rice and single late rice in Yan *et al*.^34^ for middle rice and single late rice in our study. For Chongqing, Sichuan, Guizhou, and Yunnan (AEZ 6B regions), we adopted the emission factors of middle rice in Yan *et al*.^34^ for early rice and double late rice in our study.

### Cropland change

According to FAO^20^, CO_2_ emissions from cropland change were calculated as in Eqs. (4) and (5):

4 $${E}_{i,j}^{LAND}=\mathop{\sum }\limits_{l=1}^{8}\Delta C{A}_{i,j,l}\times E{F}_{j,l}^{LAND}$$

5 $$E{F}_{j,l}^{LAND}={f}_{j,old}^{bio\& soil}-{f}_{j,new}^{bio\& soil}$$

where ${E}_{i,j}^{LAND}$ is cropland change GHG emissions from province *i* in year *j*; ∆*CA*_*i,j,l*_ is the *l* type change of cropland area from year *j* − 1 to year *j*; $E{F}_{j,l}^{LAND}$ is the emission factor of cropland change type *l* in the corresponding year; ${f}_{j,old}^{bio\& soil}$ is the carbon content in the soil and biomass in land use type before the change in the corresponding year.; and ${f}_{j,new}^{bio\& soil}$ is the carbon content in the soil and biomass in land use type after the change in the corresponding year.

The present study considered 8 types of cropland use change, which are land-use changes between cropland and wood, grassland, water and residential areas, and others. The land-use remote sensing data in 1980, 1990, 2000, 2010, and 2015 were acquired from Data Center for Resources and Environmental Sciences, Chinese Academy of Sciences (RESDC)^35^. The annual area of cropland change was calculated as the 10-year or 5-year average of the area of cropland change. The annual area of cropland change of 1978 and 1979 was assumed to be the same as that in 1980, and the annual area of cropland change of 2015 and 2016 was assumed to be the same as that in 2014.

Carbon content values for biomass of cropland, biomass and soil of wood, grassland, and others were obtained from Burney *et al*.^12^, which assumed consistent values from 1978 to 2016. According to Burney *et al*.^12^, this study assumed 70% of cropland was for crops, 15% was for oil crop plantations, and 15% was for fruits. The carbon content in biomass of cropland was selected as 10.075 t C/ha, which is 50% of the weighted average of global peak values of crops, oil crop plantations, and fruits. For wood, biomass carbon was 116.2 t C/ha, and soil organic carbon was 97.6 t C/ha, which were calculated from the mean value of the tropical evergreen forest, tropical deciduous forest, temperate broadleaf evergreen forest, temperate needle leaf evergreen forest, temperate deciduous forest, boreal evergreen forest, boreal deciduous forest, evergreen/deciduous mixed forest, dense shrubland, and open shrubland in Burney *et al*.^12^. Carbon content in water and residential areas was 0 t C/ha. For others, biomass carbon was taken as 11.5 t C/ha, and soil organic carbon as 14.5 t C/ha, the mean value of savanna, tundra, desert and polar desert/rock/ice in Burney *et al*.^12^. For soil organic carbon in cropland, the study obtained soil organic carbon in cropland in 1980 and its annual change rate during 1980–2009 in China from Yu *et al*.^36^. The annual change rates in 1978 and 1979 were assumed to be the same as that in 1980, and the annual change rates during 2010–2016 were assumed to be the same as that in 2009. Then the soil organic carbon in cropland for each year was calculated based on the above values.

### Cropland emissions

According to the People’s Republic of China Second National Communication on Climate Change^16^, the study adopted IAP-N model^21,22^ to calculate direct N_2_O emissions from cropland, and calculated indirect N_2_O emissions from cropland based on IPCC Guidelines^14^.

Eqs. (6) to (11) were used to calculate direct emissions from cropland.

6 $${E}_{i,j}^{CRLA,d}=Nit{r}_{i,j}\times E{F}_{i}^{CRLA,d}$$

7 $$Nit{r}_{i,j}=NFer{t}_{i,j}+Man{u}_{i,j}+RCro{p}_{i,j}$$

8 $$Man{u}_{i,j}=\left(1-{f}^{enn}\right)\times \mathop{\sum }\limits_{a=1}^{6}\left[Amo{u}_{i,j,a}\times {f}_{a}^{excr}\times \left({f}_{a}^{mana}+{f}_{a}^{liq}+{f}_{a}^{sol}\right)\right.$$

9 $$RCro{p}_{i,j}=\mathop{\sum }\limits_{s=1}^{11}\left(NRoo{t}_{i,j,s}+NStr{a}_{i,j,s}\times {f}^{retu}\right)$$

10 $$NRoo{t}_{i,j,s}=Pro{d}_{i,j,s}\times {f}_{s}^{dry}\times \frac{{f}_{s}^{rb}}{{f}_{s}^{harv}}\times {f}_{s}^{rbrate}$$

11 $$NStr{a}_{i,j,s}=Pro{d}_{i,j,s}\times {f}_{s}^{dry}\times \left(\frac{1}{{f}_{s}^{harv}}-1\right)\times {f}_{s}^{rbrate}$$

where ${E}_{i,j}^{CRLA,d}$ is direct cropland GHG emission from province *i* in year *j*; *Nitr*_*i,j*_ is the nitrogen input in the corresponding province and year, including the application of synthetic nitrogen fertilizer (*NFert*_*i,j*_), application of manure (*Manu*_*i,j*_), and crop residue on cropland (*RCrop*_*i,j*_), which were calculated through Eqs. (8) to (11); $E{F}_{i}^{CRLA,d}$ is the direct emission factors in the corresponding province. In Eq. (8), *f* ^*enn*^ is the fraction of NH_3_ and NOx emissions from animals or human excreta; *Amou*_*i,j,a*_ is the population of creature type *a* in the corresponding province and year; ${f}_{a}^{excr}$ is the excretion rate of manure N by corresponding creature type; ${f}_{a}^{mana}$ is the partitioning factor of excreta to anaerobic manure management systems of corresponding creature type; ${f}_{a}^{liq}$ is the partitioning factor of excreta to liquid manure management of corresponding creature type; ${f}_{a}^{sol}$ is the partitioning factor of excreta to solid storage and drylot systems of corresponding creature type. In Eq. (9), *NRoot*_*i,j,s*_ is the amount of nitrogen in crop roots of crop type *s* in province *i* in year *j*; *NStra*_*i,j,s*_ is the amount of nitrogen in crop straw and stubble of corresponding crop type, province, and year; ${f}_{s}^{retu}$ is the fraction of residue (including stubble) incorporation or return of corresponding crop type. In Eqs. (10) and (11), *Prod*_*i,j,s*_ is the crop production of crop type *s* in province *i* in year *j*; ${f}_{s}^{dry}$ is the fraction of dry matter of corresponding crop type; ${f}_{s}^{rb}$ is the shoot-to-root ratio of corresponding crop type; ${f}_{s}^{harv}$ is the crop harvest index of corresponding crop type; ${f}_{s}^{rbrate}$ is the nitrogen fraction in root biomass of the corresponding crop type.

The rural population, livestock population, and crop production were collected from annual data from the NBSC^5^. Application of synthetic nitrogen fertilizers (effective component) during 1979–1986 was collected from the Fifty Year Agricultural Statistics^37^, and the data during 1987–2016 were collected from annual data from the NBSC^5^. For poultry population data, the data during 1978–2008 were obtained from the Fifty Year Agricultural Statistics^37^, and data during 2009–2016 were obtained from the China Agriculture Statistical Report^38^. Parameters used for calculating nitrogen input were obtained from Zheng *et al*.^21^. Emission factors were obtained from Zheng *et al*.^39^ and this study adopted the mean value of all types of cropland in each region.

Eqs. (12) to (14) were used to calculate indirect emissions from cropland.

12 $${E}_{i,j}^{CRLA,ind}=Atmo{s}_{i,j}+Leac{h}_{i,j}$$

13 $$Atmo{s}_{i,j}=\left[NFer{t}_{i,j}\times {f}^{Fvolat}+Man{u}_{i,j}\times {f}^{Ovolat}\right]\times E{F}^{CRLA,atm}$$

14 $$Leac{h}_{i,j}=Nit{r}_{i,j}\times {f}^{leach}\times E{F}^{CRLA,leach}$$

where ${E}_{i,j}^{CRLA,ind}$ is indirect cropland emissions from province *i* in year *j*; *Atmos*_*i,j*_ is the amount of N_2_O-N produced from atmospheric deposition of N volatilized from managed soils in corresponding province and year; *Leach*_*i,j*_ is the amount of N_2_O-N produced from leaching and runoff of N additions to managed soils in regions where leaching or runoff occurs in the corresponding province and year. In Eq. (13), *NFert*_*i,j*_ and *Manu*_*i,j*_ are the same as direct cropland emissions; *f* ^*Fvolat*^ is the fraction of synthetic fertilizer N applied to soils; *f* ^*Ovolat*^ is the fraction of synthetic fertilizer N that volatilizes as NH_3_ and NO_x_; *EF*^*CRLA,atm*^ is the emission factor of atmospheric deposition. In Eq. (14), *Nitr*_*i,j*_ is the same as direct cropland soils; *f* ^*leach*^ is the fraction of all N added to or mineralized in managed soil in regions where leaching or runoff occurs that is lost through leaching and runoff; *EF*^*CRLA,leach*^ is the emission factor of leaching and runoff and the study adopted parameters in the IPCC Guidelines^14^.

### Machinery use

Following the method introduced by FAO^20^, CO_2_ emissions from agricultural machinery were calculated as in Eqs. (15, 16, 17):

15 $${E}_{i,j}^{ENER}=\mathop{\sum }\limits_{e=1}^{9}\left(E{C}_{i,j,e}^{plat}\times {f}_{e}^{LCV}\times E{F}_{j,e}^{ENER}\right)$$

16 $$E{C}_{i,j,e}^{plat}=E{C}_{i,j,e}^{agri}\times {f}_{i,j}^{pGDP}$$

17 $$E{F}_{e}^{ENER}={f}_{e}^{carb}\times {f}_{e}^{oxid}\times \frac{44}{12}$$

where ${E}_{i,j}^{ENER}$ is agricultural machinery GHG emissions from province *i* in year *j*; $E{C}_{i,j,e}^{plat}$ is the consumption of energy type *e* in crop farming in the corresponding province and year, which was calculated from energy consumption (physical quantity) from generalized agriculture (i.e., cropping system, livestock, fishery, and forestry) ($E{C}_{i,j,e}^{agri}$) and GDP ratio of narrow agriculture (i.e., cropping system) to generalized agriculture (${f}_{i,j}^{pGDP}$); ${f}_{e}^{LCV}$ is the average low calorific value of corresponding energy type, which was adopted to transfer energy consumption from physical quantity to standard quantity; $E{F}_{j,e}^{ENER}$ is the emission factors of corresponding energy type and year, and emission factors of primary energy were calculated from carbon content (${f}_{e}^{carb}$) and carbon oxidation factor (${f}_{e}^{oxid}$) according to IPCC Guidelines^14^.

The present study considered 9 energy types, which are coal, coke, gasoline, kerosene, diesel, fuel oil, LPG, natural gas, and electricity. Energy consumption from generalized agriculture and average low calorific value were collected from the China Energy Statistical Yearbook (CESY)^40^. Since the energy consumption of narrow agriculture was unavailable, we multiplied the energy consumption of the generalized agriculture with the GDP ratios of narrow agriculture to estimate the values (see Eq. 16). GDP ratios of narrow agriculture (i.e., cropping system) to generalized agriculture were calculated from annual data from the NBSC^5^. Carbon content and carbon oxidation factors of primary energy were obtained from Provincial Guidelines for Compiling Greenhouse Gas Emission Inventories (Trial)^41^. The emission factors of electricity for different years were acquired from the Energy Research Institute (ERI) of the National Development and Reform Commission (NDRC).

### Nitrogen fertilizer production

The study applied Eq. (18) to calculate synthetic nitrogen fertilizer production emissions, which include GHG emissions from energy mining and transportation, ammonia synthesis, fertilizer manufacture, and fertilizer transportation.

18 $${E}_{i,j}^{FERT}=NFer{t}_{i,j}\times E{F}^{FERT}$$

where ${E}_{i,j}^{FERT}$ is nitrogen production GHG emissions from province *i* in year *j*; *NFert*_*i,j*_ is the application of synthetic nitrogen fertilizers, which is the same as cropland emissions; *EF*^*FERT*^ is the emission factor of nitrogen production. The emission factor was calculated from total emissions from nitrogen fertilizer production in 2005 calculated by Zhang *et al*.^42^ and nitrogen fertilizer production in 2005 published in the 12^th^ Five-Year Plan of the Fertilizer Industry^43^.

### Pesticide production

Eqs. (19) and (20) were adopted to calculate pesticide production emissions, which include GHG emissions from pesticide manufacture and transportation.

19 $${E}_{i,j}^{PEST}=Pes{t}_{i,j}\times {f}_{j}^{plPest}\times E{F}^{PEST}$$

20 $${f}_{j}^{plPest}=\frac{Pes{t}_{j}^{pure}}{Pes{t}_{j}},\,j=2010,\ldots ,2014$$

where ${E}_{i,j}^{PEST}$ is pesticide production GHG emissions from province *i* in year *j*; ${f}_{j}^{plPest}$ is the proportion of effective component in pesticides in the corresponding year, which is defined by the ratio between the application of effective component in pesticides ($Pes{t}_{j}^{pure}$) and the application of the total amount of pesticides (*Pest*_*i,j*_); *EF*^*PEST*^ is the emission factor of pesticide production.

This study only calculated emissions from pesticide production after 1990, when the Chinese government started to publish data on pesticide use. The total amount of pesticide application was collected from annual data from the NBSC^5^. We calculated the proportion of effective components in pesticides during 2010–2014 with the application of effective components during 2010–2014 from the National Agriculture Technology Extension Service Center^44^ and the total amount of pesticide application during this period from annual data from the NBSC^5^. Then we regressed the proportion of effective components on pesticides on year to get the proportion for 1990–2009 and 2015–2016. The emission factor of pesticide production was calculated using the mean value of emission factors of all types of pesticides in Zhang *et al*.^45^.

## Data Records

**Fig. 2:**
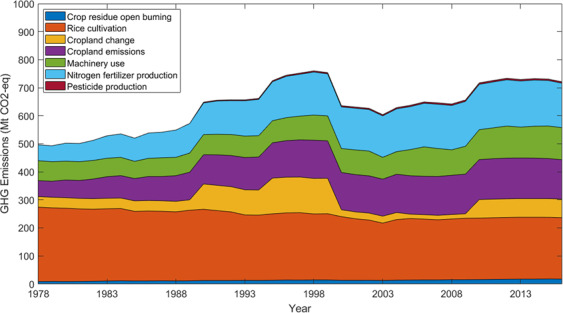
China’s GHG emissions from cropping systems, 1978–2016, in Mt CO_2_-eq. The stack area chart represents GHG emissions from 7 agricultural activities.

The dataset is available on figshare^46^. It contains one national inventory by agricultural activity and inventories by province. For the national inventory by agricultural activity, one matrix shows point estimates with 39 rows representing years and 8 columns representing agricultural activities and total emissions, while the other matrix shows 95% confidence intervals. For the inventories by province, one matrix shows total agricultural emissions with 39 rows representing years and 31 columns representing provinces, and the other seven matrixes show point estimates for emissions in 7 agricultural activities. Figure 2 presents GHG emissions by agricultural activities in China during 1978–2016.

In 2012, the total emissions from crop residue open burning, rice cultivation, and cropland emissions in the crop system were 382.59 Mt CO_2_-eq; contributing 41% of GHG emissions in agriculture (938 Mt CO_2_-eq) and 3.4% of total GHG emissions (11320 Mt CO_2_-eq) in China^8^.

## Technical Validation

### Uncertainty analysis

Uncertainty mainly comes from measurement errors of activity data, emission factors, and other parameters (see Table 1), and missing data. We evaluated particular uncertainty from emission factors and other related parameters shown in Table 1 through Monte-Carlo simulation. Normal distributions were assumed for selected emission factors and parameters in crop residue open burning, rice cultivation, cropland change, machinery use, nitrogen production, and pesticide production. Coefficients of variations (CV) for crop residue open burning were derived from existing literature, CVs for rice cultivation were from Yan *et al*.^34^, CVs for cropland change were from Burney *et al*.^12^, and CVs for machinery use were from Shan *et al*.^47^. CVs for emission factors of electricity in machinery use and emission factors in nitrogen fertilizer production and pesticide production were assumed to be 50% due to high uncertainty, according to Zhao *et al*.^48^. For cropland emissions, normal distributions and triangular distributions were assumed, where CVs and parameters for distribution were derived from Zheng *et al*.^39^ and IPCC Guidelines^14^. CVs and parameters for distribution are shown in Table 1. The activity data was adopted from government official sources, which is assumed to be subject to little/small uncertainty. The portion of missing data is small, and their uncertainty was not quantified here.

**Fig. 3:**
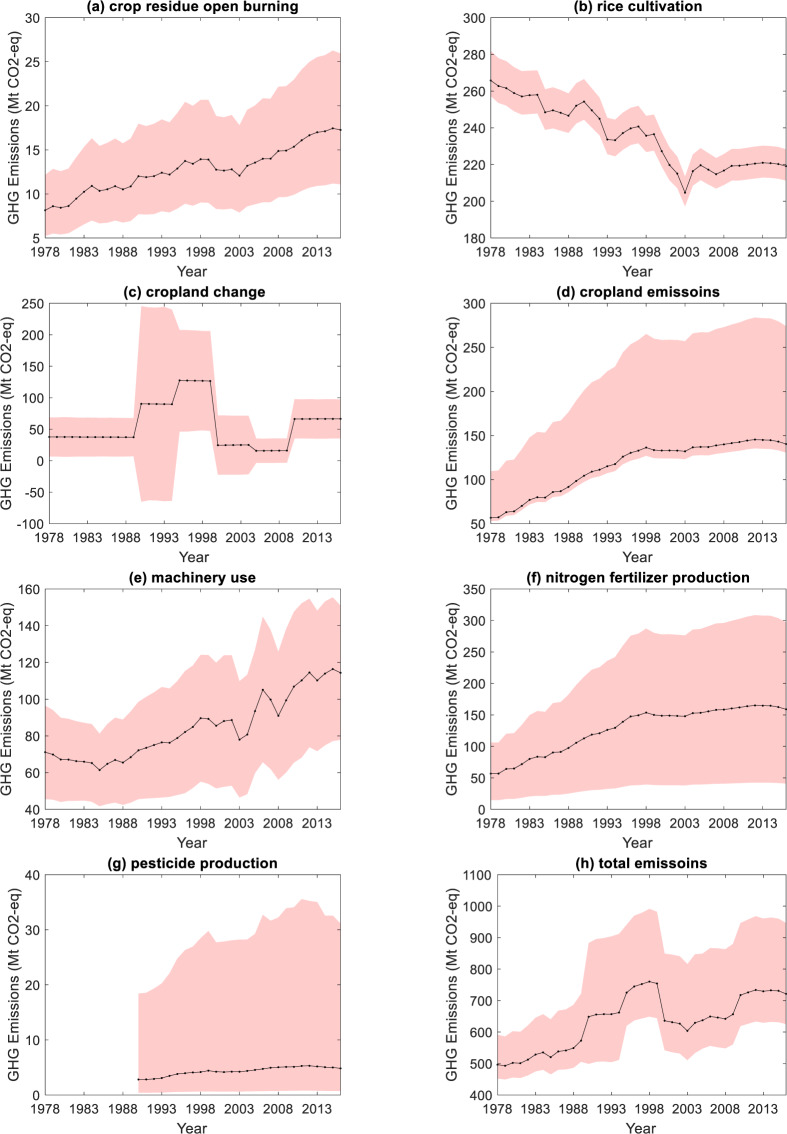
Uncertainties of the GHG emissions from cropping system in China. The graphs show the uncertainties from (**a**) crop residue open burning, (**b**) rice cultivation, (**c**) cropland change, (**d**) cropland emissions, (**e**) machinery use, (**f**) nitrogen fertilizer production, (**g**) pesticide production, and (**h**) total emissions. The lines represent the point estimates of agricultural emissions. The red area represents the 95% confidence interval of the agricultural emissions estimation.

We generated 50,000 random samples of those parameters and gained 95% confidence interval for national GHG emissions from each agricultural activity. The 95% confidence intervals of total emissions and emissions from each agricultural activity are shown in Fig. 3. For 2016, uncertainties of total emissions at 95% confidence interval are (−12%, 30%), and uncertainties of agricultural activities are (−36%, 50%) for crop residue opening burning, (−4%, 4%) for rice cultivation, (−47%, 46%) for cropland change, (−7%, 96%) for cropland emission, (−32%, 32%) for machinery use, (−73%, 87%) for nitrogen fertilizer production, and (−85%, 544%) for pesticide production.

### Limitations

Our analysis may have the following limitations: First, activity level data on cropland change adopted in this study are only available for 1980, 1990, 2000, 2010, and 2015, and we used numerical interpolation for other years. Incorporation with multi-dimensional data in interpolation in future studies may enhance continuity. Second, although the study considered the spatial heterogeneity of emission factors in crop residue open burning, rice cultivation, and cropland emissions and temporal heterogeneity of emission factors in cropland change, machinery use, and pesticide production, it applied national emission factors for cropland change, machinery use, nitrogen fertilizer production, and pesticide production, and uniform emission factors for 39 years in crop residue open burning, rice cultivation, cropland emission, nitrogen fertilizer production, and pesticide production. Further research using emission factors specific for province and year can narrow down spatial and temporal uncertainty. Lastly, this study used energy consumption in crop farming and effective components in pesticide applications derived from the GDP ratio rather than actual statistics. Uncertainty can be further reduced with more specific energy consumption data from agricultural machinery use and the number of effective components in pesticides applied to the field.

## Supplementary information

Supplementary information

## Data Availability

Matlab program is used to conduct emissions calculation and Monte-Carlo simulation. Sample codes for calculating GHG emissions from rice cultivation are available on the open-access online platform figshare^46^.
