# Author Correction: China’s greenhouse gas emissions for cropping systems from 1978–2016

**DOI:** 10.1038/s41597-023-02027-z

**Published:** 2023-03-06

**Authors:** Dijuan Liang, Xi Lu, Minghao Zhuang, Guang Shi, Chengyu Hu, Shuxiao Wang, Jiming Hao

**Affiliations:** 1 Beijing Laboratory of Environmental Frontier Technologies, School of Environment, Tsinghua University, Beijing, 100084 China; 2 State Key Joint Laboratory of Environment Simulation and Pollution Control and State Environmental Protection Key Laboratory of Sources and Control of Air Pollution Complex, Tsinghua University, Beijing, 100084 P. R. China; 3 College of Resources and Environmental Sciences; National Academy of Agriculture Green Development, Key Laboratory of Plant-Soil Interactions of MOE, China Agricultural University, Beijing, P.R. China; 4 Department of Environmental Health, Harvard T.H. Chan School of Public Health, Boston, MA USA; 5 School of Computer Science, China University of Geosciences, Wuhan, 430074 China

**Keywords:** Agriculture, Climate change, Agriculture, Climate change

Correction to: *Scientific Data* 10.1038/s41597-021-00960-5, published online 13 July 2021.

The following errors were present in the original Data Descriptor

The colours in Figure 2 on the stacked area did not correspond to the correct agricultural activities on the legend.

Table S11 (supplementary information) contained incorrect numbers for pesticide production (column 8) and the total emissions (column 9)

Figure S1 (supplementary information) contained incorrect numbers for pesticide production and the total emissions, and incorrect colours on the stacked area.

All aspects have been corrected in the pdf and HTML versions of the paper, and the supplementary information file.

## Supplementary Information

Updated supplementary file

