# Supplementary material for: China’s greenhouse gas emissions for cropping systems from 1978–2016
Source: Sci Data. 2021 Jul 13;8:171. doi: 10.1038/s41597-021-00960-5 (PMC8277801; doi:10.1038/s41597-021-00960-5)
Supplement: Supplementary file 1 — Supplementary information [file 41597_2021_960_MOESM1_ESM.docx]

**Supplementary Information**

Table of Contents

[Table S1. **Straw to grain ratio for crop types** 2](#_Toc67596842)

[Table S2. **Open burning ratio per province** 2](#_Toc67596843)

[Table S3. **Mean and coefficient of variation (CV) for length of rice-growing period and emission factor of rice types and provinces** 3](#_Toc67596844)

[Table S4. **Emission factor for cropland change from 1978 to 2016 and coefficient of variation (CV) for cropland change types** 4](#_Toc67596845)

[Table S5. **Excretion rate, partitioning factor of excreta to anaerobic manure management systems, partitioning factor of excreta to liquid manure management, and partitioning factor of excreta to solid storage and dry lot systems per creature type** 5](#_Toc67596846)

[Table S6. **Fraction of dry matter, the shoot-to-root ratio, crop harvest index, nitrogen fraction in root biomass and triangular distribution parameters per crop type** 5](#_Toc67596847)

[Table S7. **Emission factor of direct cropland emissions per province** 5](#_Toc67596848)

[Table S8. **Average low calorific value, carbon content, and carbon oxidation factor and coefficient of variation (CV) per energy type** 6](#_Toc67596849)

[Table S9. **Emission factor of electricity use from 1978 to 2016** 6](#_Toc67596850)

[Table S10. **Proportion of effective component in pesticides from 1990 to 2016** 7](#_Toc67596851)

[Table S11. **GHG emissions from cropping systems in China from 1978 to 2016 with GWP values for the 20-year horizon** 8](#_Toc67596852)

[Figure S1. **China’s GHG emissions from cropping system (using GWP_20_), 1978-2016, in Mt CO2-eq** 10](#_Toc67596853)

[References 10](#_Toc67596854)

### Table S1. **Straw to grain ratio for crop types**

| s | Crop type | $f_{s}^{str}$ | CV |
| --- | --- | --- | --- |
| 1 | rice | 0.979 | 29% |
| 2 | wheat | 1.425 | 24% |
| 3 | corn | 1.65975 | 24% |
| 4 | millet | 1.542 | 4% |
| 5 | jowar | 1.677333 | 17% |
| 6 | other cereal | 1.4 | 25% |
| 7 | beans | 1.6 | 9% |
| 8 | tubers | 0.703333 | 37% |
| 9 | cotton | 2.65325 | 26% |
| 10 | oil-bearing crops | 2 | 0% |
| 11 | fiber crops | 0.1 | 0% |
| 12 | sugar crops | 1.7 | 0% |
| 13 | tobacco | 1.058 | 0% |

* $f_{s}^{str}$ is the straw to grain ratio.

### Table S2. **Open burning ratio per province**

| i | Province | $f_{i}^{open}$ | CV |
| --- | --- | --- | --- |
| 1 | Beijing | 0.1325 | 35% |
| 2 | Tianjin | 0.1325 | 35% |
| 3 | Hebei | 0.1825 | 14% |
| 4 | Shanxi | 0.1825 | 14% |
| 5 | Inner Mongolia | 0.109 | 12% |
| 6 | Liaoning | 0.159 | 36% |
| 7 | Jilin | 0.209 | 62% |
| 8 | Heilongjiang | 0.209 | 62% |
| 9 | Shanghai | 0.3095 | 4% |
| 10 | Jiangsu | 0.2095 | 74% |
| 11 | Zhejiang | 0.3095 | 4% |
| 12 | Anhui | 0.2095 | 74% |
| 13 | Fujian | 0.3095 | 4% |
| 14 | Jiangxi | 0.1535 | 43% |
| 15 | Shandong | 0.1825 | 14% |
| 16 | Henan | 0.1825 | 14% |
| 17 | Hubei | 0.1535 | 43% |
| 18 | Hunan | 0.2645 | 34% |
| 19 | Guangdong | 0.3145 | 7% |
| 20 | Guangxi | 0.2645 | 34% |
| 21 | Hainan | 0.2645 | 34% |
| 22 | Chongqing | 0.1535 | 43% |
| 23 | Sichuan | 0.1535 | 43% |
| 24 | Guizhou | 0.1535 | 43% |
| 25 | Yunnan | 0.1535 | 43% |
| 26 | Tibet | 0.132 | 34% |
| 27 | Shaanxi | 0.1825 | 14% |
| 28 | Gansu | 0.132 | 34% |
| 29 | Qinghai | 0.132 | 34% |
| 30 | Ningxia | 0.132 | 34% |
| 31 | Xinjiang | 0.132 | 34% |

* $f_{i}^{open}$ is the opening burning ratio.

### Table S3. **Mean and coefficient of variation (CV) for length of rice-growing period and emission factor of rice types and provinces**

| i | Province | $f_{i, r}^{length}$ (d) | | | | ${EF}_{r}^{RICE}$ (mg CH_4_ /(m^2^ * h)) | | | | | |
| --- | --- | --- | --- | --- | --- | --- | --- | --- | --- | --- | --- |
|  |  | Mean | | | CV | Mean | | | CV | | |
|  |  | Early rice | Middle rice & single late rice | Double late rice |  | Early rice | Middle rice & single late rice | Double late rice | Early rice | Middle rice & single late rice | Double late rice |
| 1 | Beijing | 0 | 130 | 0 | 0% | 0 | 6.767 | 0 | 0% | 8% | 0% |
| 2 | Tianjin | 0 | 130 | 0 | 0% | 0 | 6.767 | 0 | 0% | 8% | 0% |
| 3 | Hebei | 0 | 130 | 0 | 0% | 0 | 6.767 | 0 | 0% | 8% | 0% |
| 4 | Shanxi | 0 | 130 | 0 | 0% | 0 | 6.767 | 0 | 0% | 8% | 0% |
| 5 | Inner Mongolia | 0 | 130 | 0 | 0% | 0 | 2 | 0 | 0% | 0% | 0% |
| 6 | Liaoning | 0 | 130 | 0 | 0% | 0 | 6.767 | 0 | 0% | 8% | 0% |
| 7 | Jilin | 0 | 130 | 0 | 0% | 0 | 2 | 0 | 0% | 0% | 0% |
| 8 | Heilongjiang | 0 | 130 | 0 | 0% | 0 | 2 | 0 | 0% | 0% | 0% |
| 9 | Shanghai | 77 | 110 | 93 | 0% | 8.783 | 9.283 | 13.167 | 0% | 2% | 0% |
| 10 | Jiangsu | 77 | 110 | 93 | 0% | 8.783 | 9.283 | 13.167 | 0% | 2% | 0% |
| 11 | Zhejiang | 77 | 110 | 93 | 0% | 12.35 | 9.448 | 17.983 | 15% | 0% | 13% |
| 12 | Anhui | 77 | 110 | 93 | 0% | 8.783 | 9.283 | 13.167 | 0% | 2% | 0% |
| 13 | Fujian | 77 | 110 | 93 | 0% | 12.35 | 9.448 | 17.983 | 15% | 0% | 13% |
| 14 | Jiangxi | 77 | 110 | 93 | 0% | 12.35 | 9.448 | 17.983 | 15% | 0% | 13% |
| 15 | Shandong | 0 | 130 | 0 | 0% | 0 | 6.767 | 0 | 0% | 8% | 0% |
| 16 | Henan | 77 | 110 | 93 | 0% | 8.783 | 9.283 | 13.167 | 0% | 2% | 0% |
| 17 | Hubei | 77 | 110 | 93 | 0% | 8.783 | 9.283 | 13.167 | 0% | 2% | 0% |
| 18 | Hunan | 77 | 110 | 93 | 0% | 12.35 | 9.448 | 17.983 | 15% | 0% | 13% |
| 19 | Guangdong | 77 | 110 | 93 | 0% | 12.35 | 9.448 | 17.983 | 15% | 0% | 13% |
| 20 | Guangxi | 77 | 110 | 93 | 0% | 12.35 | 9.448 | 17.983 | 15% | 0% | 13% |
| 21 | Hainan | 77 | 110 | 93 | 0% | 12.35 | 9.448 | 17.983 | 15% | 0% | 13% |
| 22 | Chongqing | 77 | 110 | 93 | 0% | 14.133 | 14.133 | 14.133 | 0% | 0% | 0% |
| 23 | Sichuan | 77 | 110 | 93 | 0% | 14.133 | 14.133 | 14.133 | 0% | 0% | 0% |
| 24 | Guizhou | 77 | 110 | 93 | 0% | 14.133 | 14.133 | 14.133 | 0% | 0% | 0% |
| 25 | Yunnan | 77 | 110 | 93 | 0% | 14.133 | 14.133 | 14.133 | 0% | 0% | 0% |
| 26 | Tibet | 0 | 130 | 0 | 0% | 0 | 2 | 0 | 0% | 0% | 0% |
| 27 | Shaanxi | 0 | 130 | 0 | 0% | 0 | 2 | 0 | 0% | 0% | 0% |
| 28 | Gansu | 0 | 130 | 0 | 0% | 0 | 2 | 0 | 0% | 0% | 0% |
| 29 | Qinghai | 0 | 130 | 0 | 0% | 0 | 2 | 0 | 0% | 0% | 0% |
| 30 | Ningxia | 0 | 130 | 0 | 0% | 0 | 2 | 0 | 0% | 0% | 0% |
| 31 | Xinjiang | 0 | 130 | 0 | 0% | 0 | 2 | 0 | 0% | 0% | 0% |

* $f_{i, r}^{length}$ is the length of the rice-growing period; ${EF}_{r}^{RICE}$ is the emission factor from rice cultivation.

### Table S4. **Emission factor for cropland change from 1978 to 2016 and coefficient of variation (CV) for cropland change types**

| Year j | ${EF}_{j,l}^{LAND}$ (t CO_2_/ha) | | | | | | | |
| --- | --- | --- | --- | --- | --- | --- | --- | --- |
|  | C-W | W-C | C-G | G-C | C-RW | RW-C | C-O | O-C |
| 1978 | -630.2 | 630.2 | -91.9 | 91.9 | 153.8 | -153.8 | 58.4 | -58.4 |
| 1979 | -629.7 | 629.7 | -91.5 | 91.5 | 154.2 | -154.2 | 58.9 | -58.9 |
| 1980 | -629.3 | 629.3 | -91 | 91 | 154.6 | -154.6 | 59.3 | -59.3 |
| 1981 | -628.9 | 628.9 | -90.6 | 90.6 | 155.1 | -155.1 | 59.7 | -59.7 |
| 1982 | -628.4 | 628.4 | -90.2 | 90.2 | 155.5 | -155.5 | 60.2 | -60.2 |
| 1983 | -628.1 | 628.1 | -89.8 | 89.8 | 155.9 | -155.9 | 60.5 | -60.5 |
| 1984 | -627.7 | 627.7 | -89.4 | 89.4 | 156.2 | -156.2 | 60.9 | -60.9 |
| 1985 | -627.3 | 627.3 | -89.1 | 89.1 | 156.6 | -156.6 | 61.3 | -61.3 |
| 1986 | -626.9 | 626.9 | -88.7 | 88.7 | 157 | -157 | 61.7 | -61.7 |
| 1987 | -626.5 | 626.5 | -88.3 | 88.3 | 157.4 | -157.4 | 62.1 | -62.1 |
| 1988 | -626.1 | 626.1 | -87.9 | 87.9 | 157.8 | -157.8 | 62.5 | -62.5 |
| 1989 | -625.7 | 625.7 | -87.4 | 87.4 | 158.3 | -158.3 | 62.9 | -62.9 |
| 1990 | -625.1 | 625.1 | -86.9 | 86.9 | 158.8 | -158.8 | 63.5 | -63.5 |
| 1991 | -624.5 | 624.5 | -86.3 | 86.3 | 159.4 | -159.4 | 64.1 | -64.1 |
| 1992 | -623.9 | 623.9 | -85.7 | 85.7 | 160 | -160 | 64.7 | -64.7 |
| 1993 | -623.4 | 623.4 | -85.1 | 85.1 | 160.6 | -160.6 | 65.2 | -65.2 |
| 1994 | -622.7 | 622.7 | -84.4 | 84.4 | 161.2 | -161.2 | 65.9 | -65.9 |
| 1995 | -622 | 622 | -83.7 | 83.7 | 161.9 | -161.9 | 66.6 | -66.6 |
| 1996 | -621.3 | 621.3 | -83 | 83 | 162.6 | -162.6 | 67.3 | -67.3 |
| 1997 | -620.6 | 620.6 | -82.3 | 82.3 | 163.4 | -163.4 | 68 | -68 |
| 1998 | -619.8 | 619.8 | -81.5 | 81.5 | 164.1 | -164.1 | 68.8 | -68.8 |
| 1999 | -619 | 619 | -80.7 | 80.7 | 165 | -165 | 69.6 | -69.6 |
| 2000 | -618.1 | 618.1 | -79.8 | 79.8 | 165.8 | -165.8 | 70.5 | -70.5 |
| 2001 | -617.2 | 617.2 | -78.9 | 78.9 | 166.7 | -166.7 | 71.4 | -71.4 |
| 2002 | -616.3 | 616.3 | -78 | 78 | 167.6 | -167.6 | 72.3 | -72.3 |
| 2003 | -615.4 | 615.4 | -77.2 | 77.2 | 168.5 | -168.5 | 73.2 | -73.2 |
| 2004 | -614.6 | 614.6 | -76.3 | 76.3 | 169.4 | -169.4 | 74 | -74 |
| 2005 | -613.6 | 613.6 | -75.3 | 75.3 | 170.4 | -170.4 | 75 | -75 |
| 2006 | -612.6 | 612.6 | -74.3 | 74.3 | 171.3 | -171.3 | 76 | -76 |
| 2007 | -611.6 | 611.6 | -73.4 | 73.4 | 172.3 | -172.3 | 77 | -77 |
| 2008 | -610.7 | 610.7 | -72.4 | 72.4 | 173.3 | -173.3 | 77.9 | -77.9 |
| 2009 | -609.7 | 609.7 | -71.4 | 71.4 | 174.2 | -174.2 | 78.9 | -78.9 |
| 2010 | -608.8 | 608.8 | -70.5 | 70.5 | 175.1 | -175.1 | 79.8 | -79.8 |
| 2011 | -607.9 | 607.9 | -69.6 | 69.6 | 176.1 | -176.1 | 80.7 | -80.7 |
| 2012 | -607 | 607 | -68.7 | 68.7 | 177 | -177 | 81.6 | -81.6 |
| 2013 | -606 | 606 | -67.8 | 67.8 | 177.9 | -177.9 | 82.6 | -82.6 |
| 2014 | -605.1 | 605.1 | -66.9 | 66.9 | 178.8 | -178.8 | 83.5 | -83.5 |
| 2015 | -604.2 | 604.2 | -65.9 | 65.9 | 179.7 | -179.7 | 84.4 | -84.4 |
| 2016 | -603.3 | 603.3 | -65 | 65 | 180.6 | -180.6 | 85.3 | -85.3 |
| CV | 27% | 27% | 94% | 94% | 20% | 20% | 62% | 62% |

* ${EF}_{j,l}^{LAND}$ is the emission factor of cropland change.

* C represents cropland, W represents wood, G represents grassland, RW represents water and residential areas, O represents others. X-Y represents the change from land-use type X to land use type Y.

### Table S5. **Excretion rate, partitioning factor of excreta to anaerobic manure management systems, partitioning factor of excreta to liquid manure management, and partitioning factor of excreta to solid storage and dry lot systems per creature type**

| a | Creature type | $f_{a}^{excr}$ (kg N/yr) | $f_{a}^{mana}$ | $f_{a}^{liq}$ | $f_{a}^{sol}$ |
| --- | --- | --- | --- | --- | --- |
| 1 | Swine | 3.22 | 0.01 | 0.38 | 0.53 |
| 2 | Sheep | 0.7 | 0 | 0 | 0 |
| 3 | Cattle | 35.675 | 0.03 | 0.02 | 0.07 |
| 4 | Poultry | 0.6 | 0.01 | 0.02 | 0 |
| 5 | Horse, ass, mule, camel, goat | 9.7 | 0 | 0 | 0 |
| 6 | Rural human | 0.69 | 0 | 1 | 0 |

*$f_{a}^{excr}$ is the excretion rate; $f_{a}^{mana}$ is the partitioning factor of excreta to anaerobic manure management systems; $f_{a}^{liq}$ is the partitioning factor of excreta to liquid manure management; $f_{a}^{sol}$ is the partitioning factor of excreta to solid storage and dry lot systems per creature type.

### Table S6. **Fraction of dry matter, the shoot-to-root ratio, crop harvest index, nitrogen fraction in root biomass and triangular distribution parameters per crop type**

| s | Crop type | $f_{s}^{dry}$ | $f_{s}^{rb}$ | $f_{s}^{harv}$ | $f_{s}^{rbrate}$ | min for $f^{root}$ | max for $f^{root}$ |
| --- | --- | --- | --- | --- | --- | --- | --- |
| 1 | rice | 0.83 | 0.125 | 0.43 | 0.00753 | 0.001453 | 0.00218 |
| 2 | wheat | 0.83 | 0.2 | 0.368 | 0.00516 | 0.001164 | 0.003491 |
| 3 | corn | 0.4 | 0.17 | 0.441 | 0.0058 | 0.000737 | 0.001052 |
| 4 | millet | 0.83 | 0.22 | 0.39 | 0.00516 | 0.002086 | 0.002745 |
| 5 | jowar | 0.4 | 0.185 | 0.386 | 0.0073 | 0.000908 | 0.001891 |
| 6 | other cereal | 0.83 | 0.22 | 0.39 | 0.00516 | 0.001318 | 0.002745 |
| 7 | beans | 0.83 | 0.2 | 0.436 | 0.02284 | 0.004348 | 0.013044 |
| 8 | tubers | 0.45 | 0.05 | 0.714 | 0.00507 | / | / |
| 9 | cotton | 0.83 | 0.2 | 0.383 | 0.00548 | 0.001188 | 0.003563 |
| 10 | oil-bearing crops | 0.83 | 0.15 | 0.256 | 0.00548 | 0.001777 | 0.003553 |
| 11 | sugar crops | 0.4 | 0.155 | 0.83 | 0.005435 | 0.000131 | 0.000681 |

* $f_{s}^{dry}$ is fraction of dry matter; $f_{s}^{rb}$ is the shoot-to-root ratio; $f_{s}^{harv}$ is the crop harvest index; $f_{s}^{rbrate}$ is the nitrogen fraction in root biomass.

* $f^{root}=f_{s}^{dry}\times\frac{f_{s}^{rb}}{f_{s}^{harv}}\times f_{s}^{rbrate}$

### Table S7. **Emission factor of direct cropland emissions per province**

| i | Province | ${EF}_{i}^{CRLA,d}$ (kg N_2_O-N/ kg N) |
| --- | --- | --- |
| 1 | Beijing | 0.004833 |
| 2 | Tianjin | 0.004833 |
| 3 | Hebei | 0.004833 |
| 4 | Shanxi | 0.004833 |
| 5 | Inner Mongolia | 0.004833 |
| 6 | Liaoning | 0.0101 |
| 7 | Jilin | 0.0101 |
| 8 | Heilongjiang | 0.0101 |
| 9 | Shanghai | 0.0094 |
| 10 | Jiangsu | 0.0094 |
| 11 | Zhejiang | 0.0094 |
| 12 | Anhui | 0.0094 |
| 13 | Fujian | 0.0162 |
| 14 | Jiangxi | 0.0094 |
| 15 | Shandong | 0.0094 |
| 16 | Henan | 0.0094 |
| 17 | Hubei | 0.0094 |
| 18 | Hunan | 0.0094 |
| 19 | Guangdong | 0.0162 |
| 20 | Guangxi | 0.0162 |
| 21 | Hainan | 0.0162 |
| 22 | Chongqing | 0.0094 |
| 23 | Sichuan | 0.0094 |
| 24 | Guizhou | 0.011083 |
| 25 | Yunnan | 0.011083 |
| 26 | Tibet | 0.004833 |
| 27 | Shaanxi | 0.004833 |
| 28 | Gansu | 0.004833 |
| 29 | Qinghai | 0.004833 |
| 30 | Ningxia | 0.004833 |
| 31 | Xinjiang | 0.004833 |

***** ${EF}_{i}^{CRLA,d}$ is the emission factor of direct cropland emissions.

### Table S8. **Average low calorific value, carbon content, and carbon oxidation factor and coefficient of variation (CV) per energy type**

| e | Energy type | $f_{e}^{LCV}$ (kJ/kg) | $f_{e}^{carb}$ (t C/TJ) | $f_{e}^{oxid}$ | CV for $f_{e}^{LCV}\times{EF}_{j,e}^{ENER}$ |
| --- | --- | --- | --- | --- | --- |
| 1 | Coal | 20908 | 26.37 | 0.93 | 17.50% |
| 2 | Coke | 28435 | 29.5 | 0.93 | 3.40% |
| 3 | Gasoline | 43070 | 18.9 | 0.98 | 2% |
| 4 | Kerosene | 43070 | 19.6 | 0.98 | 1.20% |
| 5 | Diesel | 42652 | 20.2 | 0.98 | 1.30% |
| 6 | Fuel oil | 41816 | 21.2 | 0.98 | 2.20% |
| 7 | LPG | 50179 | 17.2 | 0.98 | 7.70% |
| 8 | Natural gas | 38931 | 15.3 | 0.99 | 5.50% |
| 9 | Electricity | - | - | - | 50% |

* $f_{e}^{LCV}$ is the average low calorific value; $f_{e}^{carb}$ is the emission factors of primary energy; $f_{e}^{oxid}$ is the carbon oxidation factor.

* ${EF}_{j,e}^{ENER}=f_{e}^{carb}\times f_{e}^{oxid}\times\frac{44}{12}$

### Table S9. **Emission factor of electricity use from 1978 to 2016**

| Year j | ${EF}_{e}^{ENER}$ (g CO_2_/kWh) |
| --- | --- |
| 1978 | 1042 |
| 1979 | 1008 |
| 1980 | 968 |
| 1981 | 932 |
| 1982 | 908 |
| 1983 | 877 |
| 1984 | 897 |
| 1985 | 897 |
| 1986 | 914 |
| 1987 | 923 |
| 1988 | 923 |
| 1989 | 920 |
| 1990 | 909 |
| 1991 | 925 |
| 1992 | 927 |
| 1993 | 916 |
| 1994 | 892 |
| 1995 | 884 |
| 1996 | 892 |
| 1997 | 889 |
| 1998 | 880 |
| 1999 | 874 |
| 2000 | 854 |
| 2001 | 843 |
| 2002 | 846 |
| 2003 | 855 |
| 2004 | 837 |
| 2005 | 815 |
| 2006 | 828 |
| 2007 | 804 |
| 2008 | 760 |
| 2009 | 761 |
| 2010 | 733 |
| 2011 | 740 |
| 2012 | 697 |
| 2013 | 689 |
| 2014 | 660 |
| 2015 | 636 |
| 2016 | 614 |

* ${EF}_{e}^{ENER}$ is the emission factor of electricity use.

### Table S10. **Proportion of effective component in pesticides from 1990 to 2016**

| Year j | $f_{j}^{plPest}$ |
| --- | --- |
| 1990 | 4.35866 |
| 1991 | 4.417377 |
| 1992 | 4.476094 |
| 1993 | 4.534811 |
| 1994 | 4.593528 |
| 1995 | 4.652245 |
| 1996 | 4.710962 |
| 1997 | 4.769678 |
| 1998 | 4.828395 |
| 1999 | 4.887112 |
| 2000 | 4.945829 |
| 2001 | 5.004546 |
| 2002 | 5.063263 |
| 2003 | 5.121979 |
| 2004 | 5.180696 |
| 2005 | 5.239413 |
| 2006 | 5.29813 |
| 2007 | 5.356847 |
| 2008 | 5.415564 |
| 2009 | 5.47428 |
| 2010 | 5.620844 |
| 2011 | 5.539368 |
| 2012 | 5.567509 |
| 2013 | 5.680643 |
| 2014 | 5.84379 |
| 2015 | 5.826581 |
| 2016 | 5.885298 |

* $f_{j}^{plPest}$ is the proportion of effective component in pesticides.

### Table S11. **GHG emissions from cropping systems in China from 1978 to 2016 with GWP values for the 20-year horizon (Mt CO_2_-eq)**

| Year | Crop residue open burning | Rice cultivation | Cropland change | Cropland emissions | Machinery use | N fertilizer production | Pesticides production | Total |
| --- | --- | --- | --- | --- | --- | --- | --- | --- |
| 1978 | 21.29 | 797.33 | 37.76 | 56.50 | 71.13 | 66.05 | 0.00 | 1050.07 |
| 1979 | 22.50 | 788.26 | 37.72 | 57.03 | 69.86 | 66.05 | 0.00 | 1041.42 |
| 1980 | 22.04 | 784.77 | 37.67 | 62.81 | 67.14 | 74.71 | 0.00 | 1049.15 |
| 1981 | 22.55 | 776.76 | 37.63 | 63.73 | 67.10 | 75.34 | 0.00 | 1043.10 |
| 1982 | 24.74 | 771.22 | 37.58 | 69.92 | 66.25 | 83.44 | 0.00 | 1053.16 |
| 1983 | 26.77 | 773.32 | 37.55 | 76.70 | 65.95 | 93.08 | 0.00 | 1073.36 |
| 1984 | 28.50 | 773.99 | 37.51 | 79.71 | 65.22 | 97.19 | 0.00 | 1082.12 |
| 1985 | 27.04 | 745.34 | 37.47 | 79.29 | 61.41 | 96.36 | 0.00 | 1046.91 |
| 1986 | 27.51 | 748.56 | 37.43 | 85.54 | 64.79 | 104.98 | 0.00 | 1068.80 |
| 1987 | 28.43 | 744.58 | 37.39 | 86.54 | 66.90 | 106.11 | 0.00 | 1069.95 |
| 1988 | 27.49 | 739.83 | 37.35 | 91.39 | 65.49 | 113.33 | 0.00 | 1074.87 |
| 1989 | 28.36 | 756.14 | 37.30 | 98.09 | 68.39 | 122.91 | 0.00 | 1111.18 |
| 1990 | 31.38 | 762.85 | 90.29 | 103.97 | 72.15 | 131.03 | 2.82 | 1194.49 |
| 1991 | 31.10 | 748.62 | 90.14 | 108.62 | 73.51 | 138.05 | 2.83 | 1192.87 |
| 1992 | 31.40 | 734.84 | 90.00 | 110.79 | 75.02 | 140.45 | 2.92 | 1185.42 |
| 1993 | 32.43 | 700.92 | 89.86 | 114.67 | 76.45 | 146.77 | 3.09 | 1164.19 |
| 1994 | 31.88 | 699.84 | 89.70 | 117.15 | 76.24 | 150.52 | 3.48 | 1168.81 |
| 1995 | 33.66 | 711.42 | 127.54 | 125.51 | 78.84 | 161.74 | 3.82 | 1242.53 |
| 1996 | 35.90 | 719.10 | 127.32 | 129.90 | 82.13 | 171.57 | 3.96 | 1269.88 |
| 1997 | 35.08 | 722.11 | 127.07 | 132.36 | 84.88 | 173.68 | 4.10 | 1279.28 |
| 1998 | 36.42 | 707.08 | 126.82 | 135.84 | 89.66 | 178.70 | 4.17 | 1278.69 |
| 1999 | 36.32 | 709.58 | 126.54 | 132.98 | 89.28 | 174.42 | 4.42 | 1273.54 |
| 2000 | 33.33 | 681.84 | 24.59 | 132.48 | 85.52 | 172.87 | 4.23 | 1134.86 |
| 2001 | 33.06 | 659.23 | 24.72 | 132.55 | 88.09 | 173.08 | 4.16 | 1114.89 |
| 2002 | 33.42 | 645.24 | 24.84 | 132.38 | 88.61 | 172.53 | 4.23 | 1101.25 |
| 2003 | 31.55 | 614.05 | 24.96 | 131.56 | 77.95 | 171.94 | 4.23 | 1056.24 |
| 2004 | 34.48 | 649.16 | 25.09 | 136.10 | 80.75 | 177.70 | 4.38 | 1107.66 |
| 2005 | 35.42 | 658.91 | 15.70 | 136.56 | 93.49 | 178.29 | 4.56 | 1122.93 |
| 2006 | 36.57 | 651.67 | 15.80 | 136.40 | 105.11 | 180.95 | 4.74 | 1131.24 |
| 2007 | 36.59 | 644.24 | 15.91 | 138.23 | 99.78 | 183.72 | 4.95 | 1123.42 |
| 2008 | 38.87 | 650.17 | 16.01 | 139.43 | 90.96 | 184.18 | 5.05 | 1124.67 |
| 2009 | 38.98 | 657.90 | 16.12 | 140.84 | 99.38 | 186.34 | 5.10 | 1144.66 |
| 2010 | 40.12 | 658.12 | 66.28 | 142.04 | 106.85 | 188.24 | 5.11 | 1206.76 |
| 2011 | 42.04 | 659.83 | 66.31 | 143.77 | 110.23 | 190.46 | 5.27 | 1217.91 |
| 2012 | 43.53 | 661.55 | 66.34 | 144.87 | 114.51 | 191.93 | 5.30 | 1228.03 |
| 2013 | 44.41 | 662.94 | 66.37 | 144.41 | 110.20 | 191.48 | 5.19 | 1225.00 |
| 2014 | 44.70 | 662.25 | 66.40 | 144.16 | 113.94 | 191.37 | 5.06 | 1227.88 |
| 2015 | 45.59 | 660.80 | 66.43 | 142.59 | 116.40 | 188.87 | 5.00 | 1225.68 |
| 2016 | 45.11 | 657.36 | 66.46 | 139.83 | 114.37 | 184.78 | 4.84 | 1212.75 |

* GWP_20_ for CH_4_ is 84 and GWP_20_ for N_2_O is 264^1^.


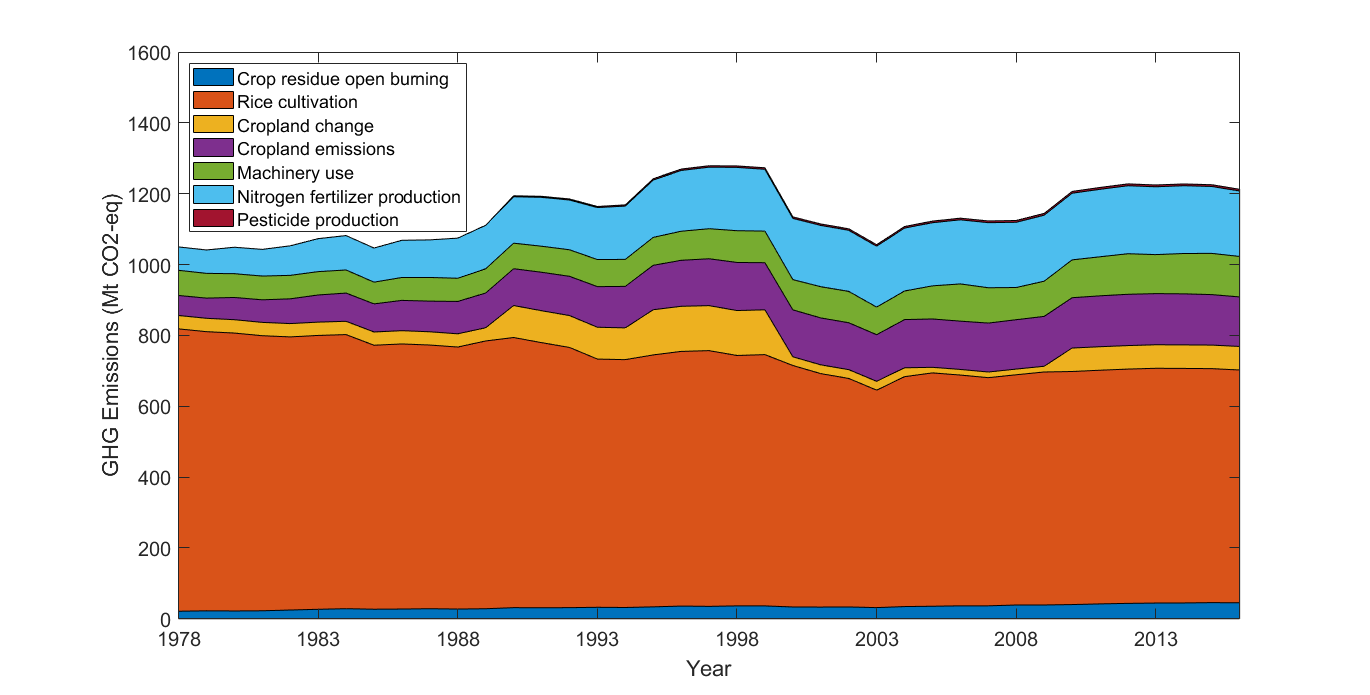


### Figure S1. **China’s GHG emissions from cropping system (using GWP_20_), 1978-2016, in Mt CO2-eq**. The stack area chart represents GHG emissions from 7 agricultural activities. GWP_20_ for CH_4_ is 84 and GWP_20_ for N_2_O is 264^1^.

## References

1. IPCC. *AR5 Climate Change 2014: Mitigation of Climate Change Summary for Policymakers and Technical Summary*. (Cambridge University Press, 2014).
